# Rapid intraoperative insulin assay: a novel method to differentiate insulinoma from nesidioblastosis in the pediatric patient

**DOI:** 10.1186/1750-1164-1-6

**Published:** 2007-10-24

**Authors:** Vivian E Strong, Alexander Shifrin, William B Inabnet

**Affiliations:** 1 Department of Surgery, Memorial Sloan-Kettering Cancer Center, New York, USA; 2 Department of Surgery, Presbyterian Hospital-Columbia University, New York, USA

## Abstract

**Introduction:**

Hyperinsulinism is the most common cause of recurrent and persistent hypoglycemia in infancy and childhood. Causes can include nesidioblastosis, pancreatic islet cell tumors such as insulinoma, and associations with multiple endocrine neoplasia syndromes. Although new, improved imaging techniques have allowed for more precise preoperative localization of insulinomas, the differentiation of nesidioblastosis and insulinoma, particularly in children, can be challenging. To improve intraoperative localization and confirmation of successful resection of insulinoma, a novel hormonal assay, the rapid intraoperative insulin assay, is reported for the first time in a pediatric patient. This intraoperative radioimmunoassay for insulin yields results within several minutes and confirms complete resection of insulinoma.

**Case description:**

We present a case of pancreatic insulinoma in a child with symptoms of severe hypoglycemia, causing seizures. The insulinoma was enucleated laparoscopically, and rapid intra-operative insulin assay used to determine the success of the procedure.

**Discussion and evaluation:**

This rapid intra-operative test provides a valuable adjunct for determining complete excision in complicated cases of recurrent or questionable insulinoma. Although not a common problem, for pediatric patients in whom the diagnosis is not clear, this test may provide a novel approach to confirming disease.

**Conclusion:**

We propose the use of this assay in facilitating intra-operative resection and confirmation of complete excision in pediatric patients. This population may especially benefit from this novel assay to confirm complete resection and to differentiate multiple etiologies of hyperinsulinism.

## Background

Hyperinsulinism is the most common cause of recurrent and persistent hypoglycemia in infancy and childhood [1]. Causes can include nesidioblastosis, pancreatic islet cell tumors such as insulinoma, and associations with multiple endocrine neoplasia syndromes. Pancreatic islet cell tumors have an annual incidence of 1 in 1,000,000, with insulinoma being the most common type, accounting for 70% to 80% of all pancreatic endocrine tumors. Peak incidence is in the third and fifth decades of life, but it can occur in all age groups. These tumors are usually solitary, benign, and diagnosed while still small because of clinical presentation of uncontrollable hypoglycemia [2]. Insulinoma is equally distributed throughout the whole pancreatic gland. Preoperative localization is sometimes challenging, with up to 64% not localized at laparotomy [3]. New improved imaging techniques have allowed for more precise preoperative localization. Noninvasive imaging modalities include transabdominal ultrasound (US), computed tomography (CT), and magnetic resonance imaging (MRI). While transabdominal US shows 30% sensitivity in detecting a tumor, conventional CT provides 73% sensitivity [4,5]. Invasive imaging procedures such as angiography and transhepatic venous hormone sampling are more accurate in detecting tumor, but they are more expensive and depend on the experience of the person performing the study. Sensitivity with angiography is 35% to 65%, and 77% to 100% with transhepatic portal venous sampling [5,6]. Selective arterial calcium stimulation and hepatic venous sampling is another invasive method, with a sensitivity of nearly 100% [6,7]. Endoscopic ultrasound (EUS) is the procedure of choice for tumor localization. With 2–3-mm resolution, EUS can be used with radial (diagnostic) or linear (for fine needle aspiration) echoendoscope and allows continuous imaging technique. Accuracy of EUS is 88% and, in combination with biphasic helical CT, increases to 97%. Intraoperative EUS can successfully localize tumors in 90% of cases [8] and can determine tumor size and invasion into the duodenum as well as vascular invasion (portal vein, small mesenteric artery, splenic vein) metastasis into the regional lymph nodes. It may distinguish nonmalignant lesions from pancreatic carcinomas.

Even with these diagnostic studies, localization of insulinoma can be difficult, and, especially in the child, must be differentiated from nesidioblastosis. This clinical and pathologic disease involves hyperplasia of the beta cells of the pancreas, which leads to persistent hyperinsulinemic hypoglycemia of infancy (PHHI) and can present in adults with organic hyperinsulinism [9]. In infants, the annual incidence is 1 in 50,000 births for the sporadic form, but may be up to 1 in 2,500 births in certain regions [10]. Treatment varies significantly, depending on symptoms, from medical management with diazoxide and chlorothiazide, nifedipine, glucagon, and octreotide to some who require partial or subtotal pancreatectomy [11]. Surgery, however, results in high rates of pancreatic endocrine and exocrine insufficiency, with long-term complications of diabetes mellitus or malnutrition.

To improve intraoperative localization and confirmation of successful resection, a novel hormonal assay, the rapid intraoperative insulin assay, has been developed. This intraoperative radioimmunoassay for insulin yields results within several minutes and confirms complete resection of insulinoma. It determines the level of active insulin molecule in the serum and is checked before insulinoma enucleation and right after enucleation intraoperatively. Although there is little guidance as to precise criteria for gauging success, one proposed protocol is similar to that used for quick parathyroid hormone (PTH) assay (i.e., a 50% drop). When insulin levels drop more than 50% of baseline within several minutes of insulinoma resection, the surgery is considered successful.

Here we present a case of pancreatic insulinoma in a child with symptoms of severe hypoglycemia, causing seizures. The insulinoma was enucleated laparoscopically – a well-accepted technique for appropriately selected lesions [8-10]. Intraoperative insulin measurements confirmed complete tumor resection. We aim to demonstrate that for the pediatric patient, in particular, the rapid intraoperative insulin assay has many advantages for the surgeon in confirming successful resection of insulin-producing tumors.

## Case presentation

The patient is a 13-year-old male with no prior medical history who presented in December of 2004 with a general tonic-clonic seizure after a basketball game. Blood glucose was 33 mg/dL. Workup was delayed. He had a second seizure in late January 2005 associated with a glucose level less than 40 mg/dL. At that time the C-peptide level was 8.1 U/dL. The patient was admitted for a 72-hour inpatient fast in late March 2005, and had a seizure at hour 18 of the fast. His glucose was 33 mg/dL with an insulin level equal to 21 U/dL, confirming the diagnosis of insulinoma. CT scanning and MRI were performed and demonstrated a 1.8 cm mass in the neck of the pancreas. After several additional seizures he was referred to Columbia Presbyterian Hospital for evaluation and admitted from the office for glucose monitoring and preoperative preparation. The patient underwent intraoperative US followed by laparoscopic enucleation of a pancreatic islet cell tumor.

## Operative procedure

An arterial line was inserted to allow frequent glucose testing during the operative procedure. The patient's baseline glucose level was 54 mg/dL with a baseline insulin level of 125 U/dL (normal 10–86 U/dL). Glucose solution was administered intraoperatively to maintain a blood glucose level greater than 100 mg/dL. After induction of general anesthesia and positioning in the modified lithotomy position, the abdomen was insufflated to 15 mm Hg with CO_2_. Two 12 mm trocars and three 5 mm trocars were inserted and the lesser sac entered through the omentum, preserving the right gastroepiploic vessels to expose the anterior surface of the pancreas. The inferior aspect of the pancreas was then mobilized to allow inspection of the posterior surface. The splenic vein was clearly identified and dissected. The pancreas was dissected medially and a space posterior to the pancreas and anterior to the portal vein was developed. Laparoscopic US identified a hypoechoic mass in the neck of the pancreas, which was enucleated using a combination of blunt and sharp dissection techniques. The lesion was placed in a retrieval sac and extracted.

Intraoperative insulin levels of 172 U/dL were measured after initial identification of the insulinoma. At 5 and 10 minutes postexcision, values dropped to 87 U/dL and 68 U/dL, respectively, demonstrating complete resection. Total operating time was 120 minutes. Postoperatively, the patient's blood glucose increased to 130 mg/dL.

The patient developed a pancreatic fistula postoperatively, which was initially controlled via drainage catheter and conservative measures, but ultimately required re-exploration with a distal pancreatectomy and splenectomy. The patient subsequently recovered without complication and final pathology showed a 1.4 × 1.2 × 0.9 cm, well-differentiated, intermediate grade insulinoma.

## Rapid insulin assay

To determine insulin levels intraoperatively, we used the STAT-IntraOperative-System (Future Diagnostics, Arlington, MA). This is a chemiluminescence immunoassay for the quantitative determination of insulin levels in human serum and EDTA plasma. The assay uses two monoclonal antibodies against insulin. Patient samples are introduced into wells and incubated for 7 minutes. The washed wells are then placed into the STAT-Read, which calculates insulin levels by measuring emissions of light in relative light units. The time between obtaining a blood sample and reading a result is under 12 minutes.

## Discussion and evaluation

This rapid intraoperative test gives us a valuable adjunct for determining complete excision in complicated cases of recurrent or questionable insulinoma. Although not a common problem, for patients with an uncertain diagnosis, this test may provide a novel approach to confirmation. This is especially the case for the pediatric patient. In this report, we highlight the utility of the rapid intraoperative insulin assay as an effective and unique method to confirm curative resection of insulinoma in the pediatric patient, a population where other hyperinsulin-producing conditions, such as nesidioblastosis, must be considered as well. In children, the etiology of hyperinsulinism can be a diagnostic challenge. This assay may represent particular utility intraoperatively to confirm curative resection.

Rapid determination of insulin levels with detection of C-peptide is a new modality for diagnosing insulinoma. C-peptide is a 31 amino acid fragment, which is split from the proinsulin molecule to form bioactive insulin in the serum. The analysis is performed by radioimmunoassay and can be done using serum from peripheral blood in a rapid time period. Insulin has a short serum half-life of about 5 minutes; 50% is cleared by the liver and 30% by the kidneys. Several authors have reported the use of intraoperative insulin monitoring during resection of insulinomas in adults, in an attempt to improve intraoperative resection and outcome [15-19]. Although intraoperative assays for parathyroidectomy are well established and available in many centers – they are currently the most widely used intraoperative means for hormone measurement in the United States – the rapid insulin assay is more complicated, is not as widely available, and requires a dedicated team in order to ensure reproducibility and reliability. This assay is a valuable technique that, when performed well, can serve as a useful adjunct to the management and operative resection of patients with known or suspected insulinoma. Our goal is to raise awareness regarding this technique and potential applications that may be useful to the surgeon.

Treatment of insulinoma is by surgical resection [2,12]. However, the surgeon may not know preoperatively if the insulinoma is single or multiple or present in a background of islet cell hyperplasia. To answer these questions, the rapid intraoperative serum insulin assay may be useful. In this case report we have presented a child with an insulinoma for whom intraoperative insulin measurements determined completeness of excision with subsequent cure. Postoperatively, the patient remained normoglycemic and all biochemical parameters returned to normal.

## Conclusion

In conclusion, we present a confirmatory case demonstrating the utility of the rapid intraoperative insulin assay in facilitating intraoperative resection and confirmation of complete excision of insulinoma for the first time in a pediatric patient. This population may especially benefit from this novel assay to confirm complete resection and to differentiate multiple etiologies of hyperinsulinism in the pediatric population.

## Competing interests

The author(s) declare that they have no competing interests.

## Authors' contributions

Performed Surgery: VES, WBI

Preparation of Manuscript: VES, AS, WBI

Critical Review and Corrections: VES, WBI

All authors read and approved the final manuscript.
